# Independent risk factors for amputation in diabetic foot

**DOI:** 10.4103/0973-3930.43096

**Published:** 2008

**Authors:** Abolfazl Shojaiefard, Zhamak Khorgami, Bagher Larijani

**Affiliations:** Department of Surgery and Endocrinology, Shariati Hospital, Tehran University of Medical Sciences, Tehran, Iran

**Keywords:** Amputation, diabetic foot, gangrene

## Abstract

**BACKGROUND:**

Diabetic foot (DF) is the main cause of nontraumatic lower extremity amputation. Early recognition and management of risk factors for foot complications may prevent amputations and other adverse outcomes.

**MATERIALS AND METHODS:**

At our hospital we have a protocol for the management of patients hospitalized because of DF. We collected clinical and laboratory data, details of diabetes complications, and history of comorbidities in 146 patients who were admitted for management of DF to determine the risk factors of amputation (major or minor) in these patients. We divided these patients into two groups, those whose treatment included amputation and those who were treated conservatively and carried out a comparative analysis of the variables in the two groups.

**RESULTS:**

Major amputation was performed in 5.5% of the patients and minor amputation in 22.6%. Those who required amputation presented a significantly higher (*P* < 0.05) incidence of nephropathy, history of previous amputation, ischemic diabetic foot and first fasting blood glucose (FBG) > 200 mg/dl after admission. Multivariable-adjusted odds ratios in stepwise logistic regression model was 2.64 for nephropathy (95%CI: 1.06 to 6.60; *P* = 0.03); 3.03 for ischemic diabetic foot (95%CI: 1.28 to 7.18; *P* = 0.01); and 3.01 for first FBG > 200 after admission (95%CI: 1.32 to 6.83; *P*= 0.01).

**CONCLUSION:**

Nephropathy, ischemic diabetic foot, and first FBG > 200 mg/dl are independent predictors of limb amputation in patients hospitalized for DF lesions. In addition to early detection and treatment of foot lesions, early management of risk factors is also important.

## Introduction

The prevalence of diabetes worldwide was estimated to be 2.8% in 2000 and is projected to be 4.4% in the year 2030, with the total number of people with diabetes expected to rise from 171 million in 2000 to 366 million in 2030.[1] Epidemiologic studies suggest that 2.5% of diabetic patients develop diabetic foot (DF) ulcers each year and 15% develop DF ulcers during their lifetime.[2]

DF is the main cause of nontraumatic lower extremity amputations[3] and precedes 85% of the cases.[4] DF lesions are a significant health and socioeconomic problems, having adverse effects on the quality of life and imposing a heavy economic burden on the patient and the State; it can lead to prolonged hospitalization and the need for rehabilitative and home care services.[5,6]

The development of a foot ulcer is traditionally considered to result from a combination of peripheral vascular disease, peripheral neuropathy and infection.[7] More recently, some factors have been identified that are believed to increase the risk of amputation in these patients.

Early recognition and management of risk factors for foot complications may prevent amputations, especially of the major type and prevent other adverse outcomes. Ethnic differences in amputation rates have been observed.[4,8,9] According to the genetic profile and cultural features of a given population, there may be differences in the risk factor pattern of the clinical complications of diabetes.

In the West, various reports are available on the risk factors for complications of diabetes; the aim of identifying these risk factors being to develop strategies for avoiding the severely reduced quality of life following amputation.[9–13] In Iran, however, little data are available on the risk factors for amputation in DF.

The present study was undertaken to identify and quantify the risk factors for lower extremity amputation (toe or foot) in diabetic patients in Iran.

## Materials and Methods

Shariati hospital is a referral center for patients with DF. We utilised a standard protocol for the management of patients hospitalized because of DF lesions. As per our protocol, we hospitalized all patients with DF who have gangrene of part of the foot; an infected ulcer in the toe or foot that requires drainage, debridement, and antibiotic therapy; signs of cellulitis in the foot or leg; a deep ulcer, with suspected underlying osteomyelitis; and septic diabetic foot (wet and infected gangrene in a large area of the foot or purulent discharge from sinus tracts in the plantar or dorsal part of foot). Patients with a small dry ulcer in the toe or foot without infection, a superficial ulcer of the skin that could be managed in the outpatient setting, and ulcer and gangrene due to reasons other than diabetes were not included in this study.

One hundred and forty-six individuals with DF who were admitted for management of DF lesions were included in this study. All subjects were patients hospitalized in the Endocrinology and Vascular Surgery Units of Shariati Hospital over a period of 1 year (January 2004 to February 2005). We collected data about these patients and conducted a descriptive-analytic study to determine the risk factors of amputation (minor and major). All but 12 patients had unilateral involvement. In those with bilateral involvement, one foot was chosen randomly for the analysis. The ethics committee of the Medical Faculty of Tehran University of Medical Sciences reviewed and approved the study.

These patients had complete neurological examinations of the foot as well as assessments of extremity pulses for determination of the extent and type of lesions. We considered the DF as ischemic when the distal pulses in the lower extremity were absent on clinical examination; we also conducted arteriographic evaluations using CT angiography or digital subtraction angiography (DSA). In patients with chronic arterial insufficiency, who had diffuse stenosis, multiple occlusions, and absence of good runoff, arterial bypass and revascularization was not possible; patients for whom arterial bypass and revascularization was performed were not included in this study.

We considered the DF as neuropathic when there was loss of vibration sense in the foot or decreased sensation on screening with a monofilament (10-g).

Laboratory evaluation, included blood glucose (BG) at admission and first fasting blood glucose (FBG) after admission. A complete medical history was also obtained; the parameters noted included sex, age, duration of diabetes, progression of diabetes complications, foot infection, atherosclerosis risk factors, and other comorbidities. We considered nephropathy as being present when persistent albuminuria of 300 mg or more (in 24-h urine collection) was confirmed. In this group of patients with nephropathy the serum creatinine was more than 1.5 mg/dl. Retinopathy was considered to be present when simple or proliferative retinopathy was observed on fundus examination.

After taking material for culture from deep tissue and a swab test, intravenous ceftriaxone (1 g bid) plus clindamycin (600 mg tid) was administered empirically; this was changed as necessary according to the results of the culture and sensitivity tests. Drainage, debridement, and amputation for foot lesions were done as necessary. Amputation was performed in both ischemic and neuropathic groups when the foot or toe was gangrenous or septic and when the infection did not respond to drainage and antibiotic therapy. As mentioned earlier, in this group of patients with ischemic DF, CT angiography or DSA indicated that revascularization was not possible.

We continued intravenous antibiotic therapy according to the patient's condition and progress of the healing process. In patients with osteomyelitis, intravenous antibiotic therapy was given for 2–4 weeks. Osteomyelitis was confirmed by bone scan in patients with a deep ulcer when the tract extended up to the bone and when presence of bone destruction or periosteal and bone surface involvement was seen in the x-ray. After discharge from hospital we continued antibiotic therapy with oral ciprofloxacin (500 mg bid) and clindamycin (300 mg tid), for 1–2 weeks; Antibiotic therapy continued for a longer duration (for up to 2 months) for patients with osteomyelitis, with assessment of the healing process.

The 146 patients were divided into two groups: those in whom amputation was performed and those who were treated conservatively. The above mentioned variables were comparatively analyzed in relation to these two groups.

### Statistical analysis

We used SPSS, version 13.5, for the descriptive statistics to report the demographic characteristics of the groups. The Chi-square test and Student's t-test were used to compare the groups with regard to the qualitative and quantitative variables, respectively. The adjusted odds ratios with 95% confidence intervals (CIs) were calculated as an estimate of the relative risks when a statistically significant difference was found between the frequencies of the variables. We used the SAS 9.1 statistical package for doing a multivariable adjusted analysis. Variables with *P* ≤ 0.15 were entered in a stepwise logistic regression model to determine independent risk factors for DF amputation.

## Results

Seventy four percent of the 146 patients had type 2 diabetes for the past 2–40 years. Ischemic DF was detected in 47.3% and neuropathy in 44.5%. The mean length of hospitalization was 13.80 ± 6.74 days. The in-hospital mortality rate was 1.3% (*n* = 2); one patient died of renal failure and the other of heart failure. The patients' characteristics are shown in the Table 1. The mean age of the female patients (56.19 ± 9.74) was 4 years lower than that of the male patients (60.15 ± 11.72); the difference was statistically significant (*P* = 0.04).

**Table 1:** Comparison of amputated and nonamputated groups

	Amputated	Nonamputated	Total	*P* value
No (%)	42 (28.8)	104 (71.2)	146 (100)	
Age in years	59.60 ± 11.12	58.24 ± 11.18	58.65 ± 11.14	0.513
Male sex (%)	26 (61.9)	65 (62.5)	91 (62.3)	0.946
Type 2 diabetes (%)	27 (90.0)	81 (97.6)	108 (74.0)	0.116
Diabetes duration (years)	13.43 ± 8.52	13.98 ± 9.69	15.23 ± 8.63	0.754
Previous amputation (%)	12 (28.6)	15 (14.4)	27 (18.5)	0.046
Diabetes treatment (%) Insulin	12 (29.3)	22 (22.0)	34 (23.3)	0.288
Hypoglycemic agent	22 (53.7)	53 (53.0)	75 (51.4)	
Insulin and hypoglycemic agent	3 (7.3)	19 (19.0)	22 (15.1)	
Atherosclerosis risk factors (%)				
Smoking	8 (19.0)	15 (14.4)	23 (15.8)	0.487
Hyperlipidemia	10 (23.8)	28 (26.9)	38 (26.0)	0.698
Hypertension	13 (31.0)	47 (45.2)	60 (41.1)	0.113
Comorbidities (%)				
History of CABG	7 (16.7)	12 (11.5)	19 (13.0)	0.404
History of MI‵	2 (4.8)	9 (8.7)	11 (7.5)	0.512
History of IHD	19 (45.2)	34 (32.7)	53 (36.3)	0.154
History of stroke	4 (9.5)	5 (4.8)	9 (6.2)	0.280
Presence of nephropathy	16 (38.1)	19 (18.3)	35 (24.0)	0.011
Presence of retinopathy	19 (45.2)	43 (41.3)	62 (42.5)	0.667
Kind of diabetic foot (%)				
Ischemic	26 (63.4)	43 (41.7)	69 (47.3)	0.020
Neuropathic	11 (26.8)	54 (52.4)	65 (44.5)	
Ischemic and neuropathic	4 (9.8)	6 (5.8)	10 (6.8)	
First FBG post-admission (mg/dl)	228.37 ± 107.11	196.40 ± 86.89	205.76 ± 94.01	0.023
BG at admission time (mg/dl)	264.53 ± 96.00	285.71 ± 91.81	260.34 ± 92.68	0.744
Creatinine (mg/dl)	1.930 ± 2.24	1.472 ± 0.99	1.607 ± 1.48	0.221
Hospital stay (days)	15.88 ± 6.92	12.21 ± 6.40	13.28 ± 6.74	0.003
Intravenous antibiotic administration (days)	13.125 ± 7.29	8.3168 ± 6.56	11.5678 ± 6.17	0.001

CABG: Coronary artery bypass graft; MI: Myocardial infarction; IHD: Ischemic heart disease

Intravenous antibiotics were administered for a mean duration of 11.57 ± 6.17 days. Ulcer drainage and debridement was performed in 41.1% and 38.4%, respectively. Major amputation (below knee or above knee) was performed in 5.5% (*n* = 8) and minor amputation (toe or transmetatarsal) in 22.6% (*n* = 33). We performed amputation only when there was a gangrenous toe (minor amputation) or foot (major amputation). Even in cases where arterial bypass and revascularization is possible, it is necessary to amputate the gangrenous toe or foot. In the presence of neuropathy or ischemia, patients with severe infection (septic foot) that does not respond to conservative management (drainage, debridement, and antibiotic therapy) must have amputation of the infected toe or foot done if sepsis is to be prevented.

The patients who had amputations were compared with non-amputated patients. On the basis of univariate analysis, in comparison with patients treated conservatively, those whose treatment required amputation (minor or major) presented a significantly higher (*P* < 0.05) incidence of nephropathy, history of previous amputation, ischemic DF, and admission FBG > 200. The odds ratios (ORs) and *P* values are shown in Table 2.

**Table 2:** Odds ratios of different risk factors in patient with diabetic foot

	Univariate analysis			Multivariate analysis		
	OR	95% CI	*P* value	OR	95%CI	*P* value
First FBG > 200 mg/dl	2.34	1.11–4.91	0.023	3.01	1.32–6.83	0.0109
Ischemic diabetic foot	2.33	1.10–4.93	0.025	3.03	1.28–7.18	0.0101
Nephropathy	2.75	1.24–6.11	0.011	2.64	1.06–6.60	0.0345
Previous amputation	2.37	1.00–5.63	0.046

Hosmer and Lemeshow goodness-of-fit test; *P* = 0.9168 and area under ROC curve = 0.75082 in stepwise logistic regression model

The mean length of hospitalization was significantly higher in patients who underwent amputation. There was a strong correlation between duration of hospital stay and duration of antibiotic administration (r = 0.73; *P* < 0.001).

Multivariate-adjusted odds ratios in the stepwise logistic regression model was 2.64 for nephropathy (95%CI: 1.06 to 6.60; *P* = 0.03); 3.03 for ischemic diabetic foot (95%CI: 1.28 to 7.18; *P* = 0.01); and 3.01 for admission FBG > 200 (95%CI: 1.32 to 6.83; *P* = 0.01) [Table 2]. These three variables, but not history of previous amputation, appear to be independent risk factors for DF amputation. There were no significant differences between the two groups in relation to age, sex, duration of diabetes, kind of treatment, cardiovascular risk factors, retinopathy, and level of BG.

## Discussion

Results of the analysis demonstrate that nephropathy, ischemic DF, and admission FBG > 200 mg/dl, as well as history of previous amputation, are independent risk factors for amputation. This is the first study to document the independent risk factors for amputation in DF in an Iranian population.

Several risk factors for amputations among diabetics have been cited in the literature. However, there are inconsistencies between the studies. Markowitz *et al.*[10] in a retrospective case-control study observed that amputation was significantly increased by male gender, renal disease and peripheral vascular disease. Carlson *et al.*[11] demonstrated digital deformity, diabetic neuropathy, and ischemia as obvious risk factors for toe amputations. Other less obvious risk factors included gender, foot infection, foot abscess, osteomyelitis, diabetic retinopathy, and diabetic nephropathy. Chaturvedi *et al.*[9] showed that vascular complications and their risk factors are themselves risk factors for amputation in both type I and type II diabetes. They suggested elevated levels of glucose and triglyceride and presence of retinopathy as key risk factors for amputation. Miyajima *et al.*,[14] in a review of 210 diabetic patients over the past 9 years, showed that atherosclerosis obliterans with multiple stenosis, hemodialysis, and elevated HbA1C were independent risk factors for major amputation.

Thus, various risk factors have been identified by different studies. This variability may be due to variations in the study designs, as well as differences in the genetic profile and cultural features of the populations studied. Ethnic differences in amputation rates have been observed,[4,8,9] which could reflect true differences in pathophysiology but could also be a consequence of inequalities in access to health care and the degree to which risk factors for amputation are common among the population. Asian patients with diabetes mellitus are less prone to foot ulcers than Caucasians,[15] which may be related to cultural differences in self-care.[7]

Ischemic DF, with or without the presence of neuropathy and infection, was an independent risk factor for amputation in our analysis. Arterial insufficiency that did not present the possibility of revascularization because of multiple stenosis or occlusions with poor runoff, as demonstrated by clinical and arteriographic analysis, allowed us to demonstrate its significant influence on these patients' outcome. Diabetics have a high risk of atherosclerotic peripheral vascular disease.[7,16] In combination with peripheral neuropathy and minor trauma it would be a cause of foot ulceration.[7] It also has a major role in delayed wound healing and the development of gangrene.[17] The presence of peripheral arterial disease has been cited by many authors as a risk factor for amputations in diabetics.[10–14] It is irrefutable that the presence of peripheral vascular disease causes problems in the blood flow; adequate blood flow is essential for healing and for combating the severe infections that attack diabetic feet. Calle-Pascual.[18] reported that 100% of the major amputations in their series were associated with peripheral vascular disease.

Our finding that nephropathy was also an independent risk factor for amputation in DF patients is consistent with other reports.[10,11,12,14,19,20] An increased risk of DF ulcer with diabetic nephropathy was detected by the American Diabetes Association consensus group.[21] This group also showed an increased risk of nonvascular DF ulcer including 40% of the patients with chronic renal failure.[22]

The analysis also shows, that admission FBG > 200 mg/dl is another independent risk factor for amputation. Lehto *et al.*[23] reported that the risk of amputation increases in a linear fashion with increases in plasma glucose level. Boyoko *et al.* found that severe hyperglycemia was associated with a higher risk for DF ulcer.[24] Chaturvedi *et al.*[9] also suggested glucose level as a key risk factor for amputation. However, many studies have shown that levels of blood glucose over periods of time, as assessed by HbA1C, would be a better predictor for DF amputation.[12,14,19,20,25]

History of a previous amputation in either foot[13,20] may predict another amputation. However, such a previous history was not an independent risk factor according to our analysis. This could be related to the long duration of diabetes, poor control of blood glucose, poor hygiene, and presence of neuropathy and generalized atherosclerosis. Apelqvist *et al.* found a recurrence rate of DF amputation of 34% after 1 year and 70% after 5 years.[26] Although many previous studies agree that foot infection is a risk factor for DF amputation,[11,20,27,28] our data did not reveal an association; this might be a result of our management protocol, whereby we attempt to save any feet with infection by means of frequent debridement, drainage, and washing and dressing, along with antibiotic therapy and daily assessment of the healing process. We will be reporting our experience in the management of septic diabetic feet and our success in preventing amputation in these patients in another paper in the near future.

We also failed to show any association between amputation and age, sex, duration of diabetes, neuropathy and retinopathy.

DF lesions are a significant health and socioeconomic problem, having adverse effects on quality of life and imposing a heavy economic burden on the patient; it can result in prolonged hospitalization and the need for rehabilitation and home care services.[5,6] The majority (22%) of our patients had minor amputations and they could continue to walk on their feet. If we can prevent foot lesions in patients with diabetes and control the risk factors, patients will have an acceptable quality of life. To prevent amputations and the consequent adverse impact on the patient's daily activities, the first requirement is strict control of diabetes, which is the primary disease. Early detection and treatment of lesions and regular foot care is also important.

The importance of identifying these risk factors is that such knowledge is useful for developing methods to detect them at an early stage and thus prevent limb amputation. Whenever risk factors are detected, relevant advice can be given to the patient.
